# Recent Advances in Carbonaceous Photocatalysts with Enhanced Photocatalytic Performances: A Mini Review

**DOI:** 10.3390/ma12121916

**Published:** 2019-06-13

**Authors:** Jianlong Ge, Yifan Zhang, Soo-Jin Park

**Affiliations:** Department of Chemistry and Chemical Engineering, Inha University, 100 Inharo, Incheon 22212, Korea; gejianlong1121@126.com (J.G.); zyf910626@inhaian.net (Y.Z.)

**Keywords:** carbon, photocatalysts, bandgap engineering, solar energy, visible light photocatalysis

## Abstract

Photocatalytic processes based on various semiconductors have been widely utilized in different applications, with great potential for use in environmental pollution remediation and sustainable energy generation. However, critical issues, including low light adsorption capability, wide energy bandgap, and unsatisfactory physicochemical stability still seriously limit the practical applications of photocatalysts. As a solution, the introduction of carbonaceous materials with different structures and properties into a photocatalyst system to further increase the activity has attracted much research attention. This mini review surveys the related literatures and highlights recent progress in the development of carbonaceous photocatalysts, which include various metal semiconductors with activated carbon, carbon dots, carbon nanotubes/nanofibers, graphene, fullerene, and carbon sponges/aerogels. Moreover, graphitic carbon nitride is also discussed as a carbon-rich and metal-free photocatalyst. The recently developed synthesis strategies and proposed mechanisms underlying the photocatalytic activity enhancement for different applications are summarized and discussed. Finally, ongoing challenges and the developmental direction for carbonaceous photocatalysts are proposed.

## 1. Introduction 

With the rapid development of economic and industrial techniques, environmental pollution and energy shortage have emerged as worldwide issues that seriously limit the sustainable development of human society. To address these problems, the development of industrial techniques must focus on meeting the demand for clean energy and tackling environmental pollution [1]. Therefore, photocatalytic processes that can be induced by environmentally friendly and inexhaustible solar energy have attracted significant research attention owing to their several advantages, such as the renewable nature of the energy sources, safety, and low operating cost [2,3]. Until now, numerous photocatalytic processes have been developed for various applications, such as degradation of organic pollutants [4,5], water splitting [6,7], reduction of CO_2_ [8,9], bacteria disinfection [10,11], and selective synthesis of organic compounds [12,13]. Undoubtedly, in all photocatalytic processes, the photocatalyst is the core and thus, the entire performance of a photocatalytic process is mainly decided by the photocatalytic activity of the corresponding photocatalysts [2,14]. In general, photocatalysts are prepared from semiconductors, such as the most popular TiO_2_, which was first used for photocatalytic water splitting by Fujishima and Honda in the early 1970s [15]. Subsequently, numerous semiconductors were developed and utilized for photocatalysis applications. However, there are many problems associated with the current photocatalysts, such as the quite-low utilization efficiency of visible light from solar energy, wide energy bandgap, high recombination rate of photogenerated electron–hole pairs, and insufficient physicochemical stability of metal semiconductors, which seriously limit the practical application of photocatalysts [16,17,18]. Moreover, the urgent development of metal-free photocatalysts is also crucial owing to the limited storage of the related metals on the earth [19,20,21]. Therefore, it is imperative to develop next-generation photocatalysts with high visible-light efficiency, low cost, and good stability.

Thus far, a variety of strategies, such as coupling semiconductors with carbon [22,23,24], introducing co-catalysts [25,26,27], and constructing heterojunctions [28,29,30], have been developed to further improve the performance of photocatalysts. Among which, carbonaceous materials play important roles in the design and synthesis of novel photocatalysts as they have the advantages of good electron conductivity, large surface area, excellent physicochemical stability, and facile synthesis approaches [22]. In general, the micro/macro morphologies and crystal structures of carbonaceous materials could be well regulated, so that these materials can be facilely synthesized according to the specific requirements of different applications. Up to now, a great deal of photocatalysts based on carbonaceous materials with various structures and compositions have been developed. The most widely used carbonaceous materials for the synthesis of photocatalysts are activated carbons, carbon dots, carbon nanotube/nanofiber, graphene, fullerene, and three-dimensional (3D) carbons. Moreover, as a carbon-rich and metal-free photocatalyst, graphitic carbon nitride (g-C_3_N_4_) has also attracted great research attention due to its appealing bandgap property, high physicochemical stability, facile synthesis approach, and “earth-abundant” nature [31,32,33,34]. Owing to the attractive features of carbonaceous materials for photocatalytic applications, there have been many outstanding studies focusing on the development of photocatalysts deriving from carbonaceous materials [22]. Therefore, a timely summary and discussion of the recent progresses on carbonaceous photocatalysts is required. Moreover, the influence of the morphology and composition of the carbonaceous materials on the photocatalytic process must be analyzed, as this knowledge is important for the design and synthesis of new carbonaceous photocatalysts.

**Scheme 1:**
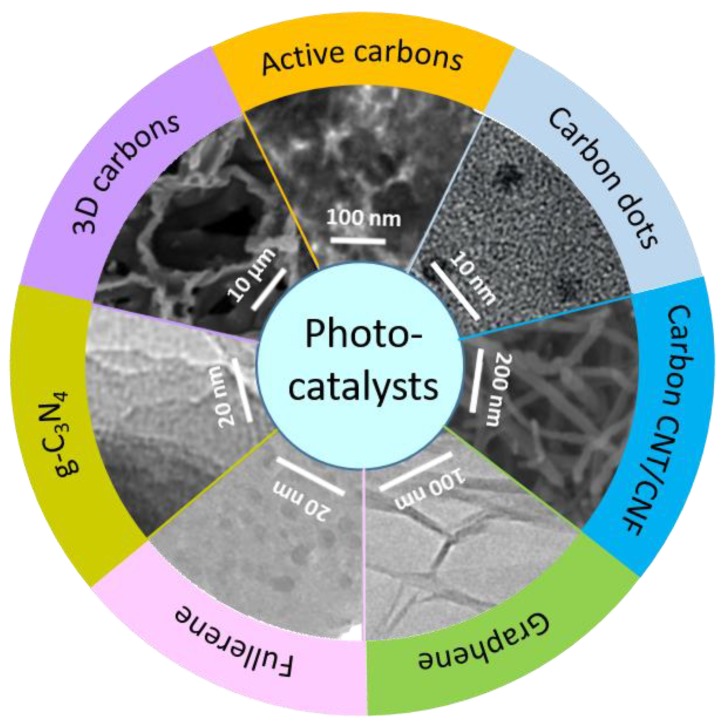
Carbonaceous materials for photocatalysts. Adapted with permission from Reference [35] Copyright (2017) Springer; [36] Copyright (2018) Wiley; [37] Copyright (2018) Springer; [38] Copyright (2018) Wiley; [39] Copyright (2019) American Chemical Society; [40] Copyright (2018) Elsevier; [41] Copyright (2019) Elsevier.

Therefore, as shown in Scheme 1 and Table 1, in this mini review we demonstrate a brief summary and a critical discussion on the recent developments of carbonaceous photocatalysts for various applications, such as the photodegradation of organic pollutants, water splitting, reduction of CO_2_, etc. In particular, much more attention has been paid to the fabrication strategies of different carbonaceous photocatalysts based on various carbonaceous materials with obviously improved visible-light activity. The demonstrated carbonaceous materials are commonly used in photocatalysis, including activated carbon, carbon dots, carbon nanotube/nanofiber, graphene, fullerene, g-C_3_N_4_, and carbon sponges/aerogels. Additionally, the proposed mechanisms of carbonaceous materials for enhancing the photocatalytic performances of the pristine photocatalysts are discussed simultaneously. Finally, the major challenges and opportunities for the development of carbonaceous photocatalysts are discussed. Specifically, we anticipate that this mini review could be helpful for the understanding of the development in the trend of the next generation of carbonaceous photocatalysts, and thus provide a guidance for the design and fabrication of novel visible-light photocatalysts with enhanced performance.

## 2. Principles of the Carbonaceous Photocatalysts

**Figure 1:**
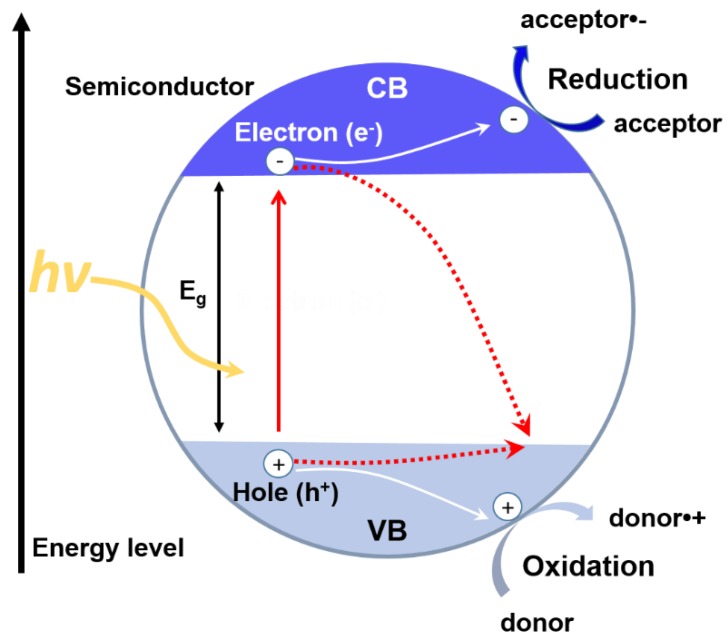
Schematic illustration demonstrating the photocatalytic process of a semiconductor photocatalysts. Adapted with permission from Reference [22]. Copyright (2011) Elsevier.

To better understand the design criteria of carbonaceous photocatalysts, the basic principle of photocatalysis is discussed in this section. As we know, the photocatalytic process is a type of advanced oxidization process (AOP), which is driven by the light energy [14,42,43]. In most cases, as the core of the various photocatalytic processes, photocatalysts are semiconductors with good light sensitivity owing to their unique electronic bandgap structure with the filled valence band (VB) and the empty conduction band (CB) [29,44,45,46]. As shown in Figure 1, when the light irradiates on the surface of a photocatalyst, part of it will be absorbed, and if the absorbed light contains photons with energy levels greater than the bandgap energy (E_b_) of the photocatalyst, electrons will generate from the VB and move to the CB, at the same time, the corresponding holes in the VB will emerge [44]. After that, the generated electron–hole pairs will migrate to the surface of photocatalyst for the redox reactions [42,47]. During the whole photocatalytic process, a series of redox reactions will occur depending on the specific applications, including oxidation induced by the photogenerated holes and reduction caused by the photogenerated electrons (Figure 1). However, during the migration process of electron–hole pairs, fast recombination will simultaneously occur, which is negative for the AOPs that are yet to be suppressed [48,49,50]. On the other hand, to effectively utilize the redox reactions, a greater number of active sites must be generated on the surface of the photocatalysts [51]. Consequently, at least five critical aspects must be satisfied to improve the photocatalytic activities of the corresponding photocatalysts: 1) reduce the E_g_ of the catalytic system to increase the visible-light utilization efficiency; 2) increase the light absorption capacity of the photocatalyst to generate more electron–hole pairs; 3) retard the electron–hole pair recombination to improve their quantum efficiency; 4) increase the surface area of the photocatalyst to provide a greater number of reaction sites, and 5) improve the physicochemical stability of the photocatalyst to ensure a durable service performance during the reaction. 

## 3. Synthesis and Applications of Carbonaceous Photocatalysts

Carbonaceous materials, especially carbonaceous nanomaterials, have attracted much research attention in the area of photocatalysis owing to their intriguing properties and good tunability by regulating their structures and compositions [17,22]. Until now, many carbon-based photocatalysts with different structures and compositions have been invented through a variety of well-designed synthesis strategies, which will be systematically summarized in this section. 

### 3.1. Activated Carbon 

Activated carbon (AC) is a well-known highly porous carbonaceous material that is widely used as an adsorbent to remove various organic compounds from both air and water because of its large surface area, good physicochemical stability, and ease of fabrication [52,53,54,55,56]. Additionally, AC can also be used as an effective support for various semiconductors to form composite photocatalysts [57,58,59,60,61]. The synergistic effects between AC and semiconductors can further enhance the photocatalytic activity. In general, the roles that AC plays in a variety of photocatalysis systems are as follows: (1) the ultrahigh surface area of AC endows the composite photocatalysts with good ability to adsorb or capture organic molecules in water or in air, thus ensuring a high concentration of organic compounds around the surface of the photocatalysts, which is critical for the improvement of photocatalytic reaction rates. (2) The intermediates generated after photocatalysis can be effectively adsorbed by AC for the subsequent degradation cycle so that the availability of the photocatalyst can be improved. (3) The combination of AC with semiconductors can partially suppress the combination of photogenerated electron–hole pairs so that the photocatalytic activity of AC/semiconductors composite systems can be improved. Consequently, AC has been considered a promising support for the design and synthesis of novel photocatalysts, and many AC/semiconductors composite photocatalysts with different properties have been synthesized through various strategies. According to its macroscopic structure, AC can be clarified as powdered AC or AC fibers. In this review, some representative AC/semiconductors photocatalysts and their fabrication strategies are discussed. 

#### 3.1.1. AC Powder

**Figure 2:**
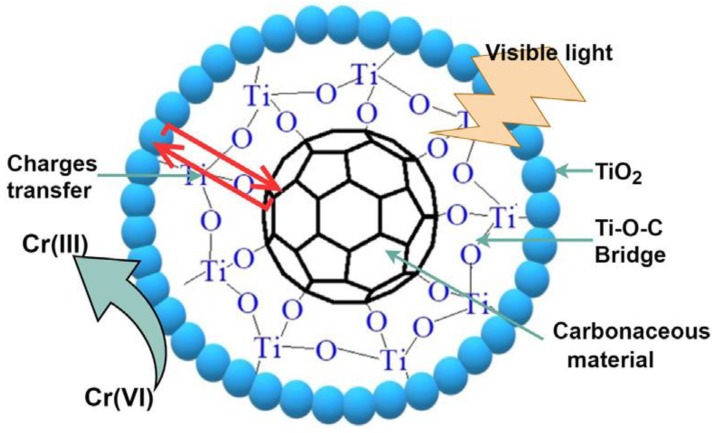
Schematic of proposed degradation of pollutants with TiO_2_/carbon composites. Adapted with permission from Reference [63]. Copyright (2019) Elsevier.

Powdered AC is the most commonly used adsorbent for environmental pollution remediation. It is believed that the strong adsorption ability of AC can lead to synergistic effects with various photocatalysts to further enhance their performance [22,24]. To date, numerous semiconductors have been coupled with powdered AC to form composite photocatalysts, among which the TiO_2_/AC composite is the most widely reported. In early years, Zhang et al. [62] claimed that a system of particles suspension was beneficial for mass transfer on the surface of the photocatalysts and for light absorption. As a result, they synthesized two kinds of TiO_2_/AC composite photocatalysts using the sol-gel method and metal organic chemical vapor deposition (MOCVD). They compared the structures and photocatalytic activities of the obtained composite photocatalysts. The results demonstrated that the TiO_2_/AC photocatalysts synthesized by these two methods both exhibited eggshell-like microstructures and similar activities. However, the composite photocatalysts made by the MOCVD method were preferred because of the formation of a TiO_2_+AC hybrid film on the surface of the AC template with the Ti–O–C bond, which could effectively recapture the intermediates generated from the degradation of organic compounds and ensure a better stability, while it could not be found in the TiO_2_/AC composite produced by the common sol-gel method. Unfortunately, the surface area of the TiO_2_/AC composite produced by MOCVD decreased due to the blockage of mesopores by TiO_2_, which hinders further enhancement of its photoactivity. To address this problem, Djellabi et al. [63] successfully synthesized a new kind of TiO_2_/AC composite photocatalyst via an ultrasonic-assisted sol-gel method followed with a simple calcination process. The structure characterization results demonstrated that a Ti–O–C bond was also formed between the TiO_2_ and the activated carbon template (Figure 2), and a large surface area was obtained due to the better dispersion uniformity of the TiO_2_ nanoparticles on the surface of the AC. Therefore, the obtained TiO_2_/AC composite photocatalyst exhibited significantly enhanced photocurrent responses to the visible light compared with the pristine TiO_2_. Nowadays, apart from the TiO_2_, a variety of newly developed semiconductors have also been coupled with powdered AC to form composite photocatalysts for different applications [58,60,61,64,65,66,67,68,69,70,71]. 

#### 3.1.2. AC Fiber

**Figure 3:**
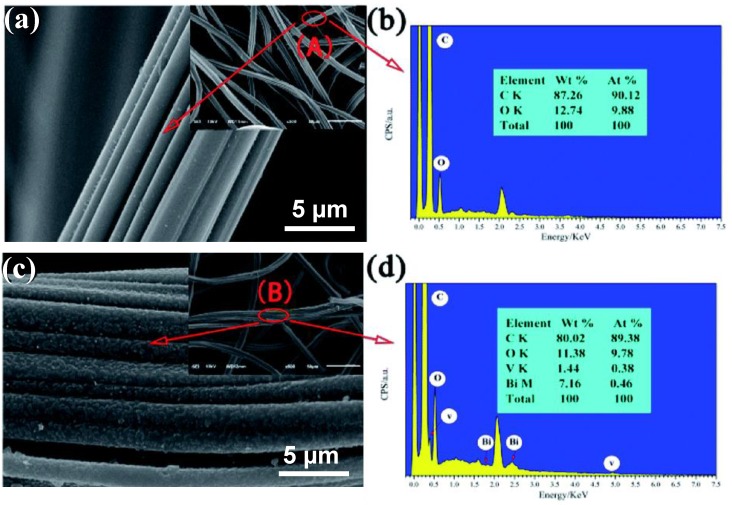
SEM images (**a**,**b**) and corresponding EDS spectra (**c**,**d**) original activated carbon fibers (ACFs) and BiVO_4_@ACFs. Adapted with permission from Reference [79]. Copyright (2018) Royal Society of Chemistry.

Similar to the powdered AC, AC fibers (ACFs) are highly porous carbon materials with a large surface area, high pore volume, and uniform pore-size distribution [24]. Consequently, ACFs have similar adsorption capacities to powdered AC, but have a better recyclability owing to their fibrous structure with a high aspect ratio, thus the ACFs can be easily recovered after use [54,72,73,74]. Currently, various ACFs are employed as templates for different semiconductors to fabricate high-performance ACF/semiconductors composite photocatalysts [35,58,75,76,77]. For example, Wang et al. [78] loaded N-doped TiO_2_ particles on the surface of ACF via a facile impregnation method and the followed heat treatment. The obtained N-doped TiO_2_/ACF retained its large surface area and exhibited enhanced visible-light photocatalytic activity. Similarly, the synergy effect between the strong adsorption performance of ACF and the improved photocatalytic property of N-doped TiO_2_ was the main reason for the enhanced photocatalytic degradation performance of the composite photocatalysts [24]. Apart from TiO_2_, other semiconductors can also be loaded on the surface of ACFs. For example, Zhang et al. [79] fabricated a BiVO_4_@ACF composite photocatalyst through a facile hydrothermal immobilization method. As shown in the scanning electron microscope (SEM) images and the corresponding energy-dispersive spectrometry (EDS) in Figure 3, the BiVO_4_@ACFs retained intact fibrous structures and good uniformity of the loaded BiVO_4_. Moreover, they found that the visible-light adsorption performance of BiVO_4_@ACFs was much greater than that of pristine ACFs and individual BiVO_4_ and they also claimed that this difference was due to the formation of a heterojunction electric field between BiVO_4_ and the ACFs. As a result, the obtained BiVO_4_@ACFs exhibited intriguing photocatalytic degradation performance for the organic compound in water. As expected, the ACFs-based composite photocatalysts demonstrated in those works exhibited durable mechanical properties and could be easily collected from the reaction solution. Consequently, ACFs can be widely utilized as the support for all kinds of semiconductors and can also be easily assembled owing to their excellent mechanical properties and good weaving performance, which is superior to the powdered ACs. 

### 3.2. Carbon Dot

**Figure 4:**
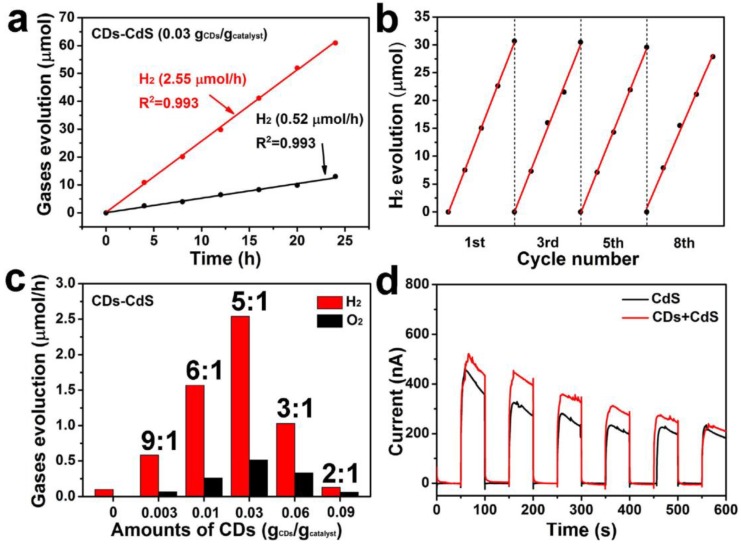
(**a**) Water splitting performance of the carbon dots (CDs)–CdS under visible light irradiation. (**b**) The cyclic stability of visible light photocatalytic water splitting performance of CDs–CdS. (**c**) The amount of produced gases of CDs–CdS with different content of CDs. (**d**) Photoresponse properties of pristine CdS and representative CDs–CdS. Adapted with permission from Reference [85]. Copyright (2017) Elsevier.

Carbon dots (CDs) are recently developed carbon nanomaterials that possess various intriguing properties such as unique fluorescence, good conductivity, good physicochemical stability, and environmental friendliness [80]. Since their invention, CDs have been widely used in various applications, among which photocatalysis is one of the most extensively researched areas [22,81,82,83]. Early on, Li et al. [84] fabricated CDs with sizes of 1.2–3.8 nm via a facile one-step alkali-assisted electrochemical method. Subsequently, a variety of photocatalysts were synthesized based on the obtained CDs, and the as-prepared composite photocatalysts (TiO_2_/CDs and SiO_2_/CDs) both showed an effective photoresponse to the full spectrum of sunlight due to the upconversion luminescence properties of the CDs. Based on these early studies, many novel photocatalysts based on CDs have been developed in recent years. For example, Zhu et al. [85] fabricated a composite photocatalyst derived from cadmium sulfide (CdS) nanosheets and CDs (~5 nm) via a simple hydrothermal method. The employed CDs in this work were synthesized by a typical electrolytic method with graphite rods as the carbon source. They found that the introduced CDs not only effectively suppressed the oxidation of CdS but also could react with CdS to form a C–S bond, which showed good inoxidizability. As a result, the photocorrosion resistance of CdS was significantly enhanced. Moreover, the CDs could further enhance the light absorption capacity and the strong contact of the CDs with CdS nanosheets ensured a fast charge transport and a high separation efficiency in addition to promoting the production of O_2_ by suppressing the combination of photogenerated holes and S^2−^ to stabilize the catalysts. These features are of great importance for the enhancement of photocatalytic performance, therefore the CDs–CdS composite exhibited an excellent photocatalytic water splitting performance with significantly enhanced stability without the requirement for any sacrificial agents or cocatalysts (Figure 4). More recently, CDs-based meta-free photocatalysts have also been developed. For example, Sarma et al. [86] fabricated a new kind of carboxyl-functionalized CDs and investigated their visible-light photocatalytic activity. The results demonstrated that the functional groups on the surface of the CDs play a critical role in photocatalytic oxidation reactions and the carboxyl groups on the surface of the CDs were effective for promoting the transport of photogenerated electrons and inhibiting electron−hole recombination. As a result, the carboxyl-functionalized CDs exhibited good visible-light-driven aerobic C−H oxidation performance toward alkyl benzenes. However, due to the nanoscale size of CDs and the zero-dimensional (0D) structure, the collection of photocatalysts after use is quite difficult, which is a critical issue that needs to be addressed for practical applications. 

### 3.3. Carbon Nanotube/Nanofiber

Carbon nanotubes (CNTs) and carbon nanofibers (CNFs) are typical one-dimensional (1D) carbon nanomaterials that possess the characteristics of both nanomaterials and fibrous structures. With their large surface area, intriguing electronic properties, unique physicochemical properties, and high aspect ratio, CNTs and CNFs are promising candidates for the design and synthesis of novel photocatalysts [87,88,89,90,91]. Nowadays, numerous studies have been carried out on the design and fabrication of CNT- and CNF-based composite photocatalysts. Herein, we will discuss some representative studies.

**Figure 5:**
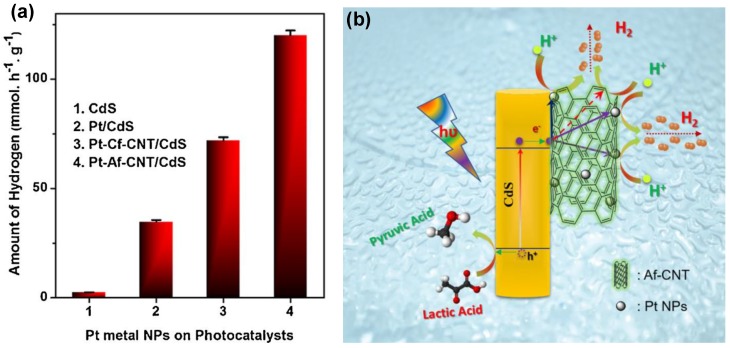
(**a**) Comparison of hydrogen production with Pt nanoparticle-loaded materials versus pure CdS. (**b**) Schematic illustration demonstrating mechanism of H_2_ production via photocatalytic water splitting by Pt–Af–CNT/CdS ternary composite. Adapted with permission from Reference [109]. Copyright (2018) Elsevier.

Owing to the facile fabrication method and relative low cost, multiwalled carbon nanotubes (MWCNTs) have been widely employed to prepare the photocatalysts [92,93]. In general, CNTs mainly act as supports for various semiconductors to form composite photocatalysts. Various synthetic approaches for CNT-based composite photocatalysts have been proposed, such as the sol-gel method [22,94,95,96,97,98], vapor phase method [99,100], chemical vapor deposition method [101,102,103], and hydrothermal method [103,104,105,106]. Among which, the sol-gel method is a common and easy strategy and therefore, it is widely used for the fabrication of CNT/semiconductor composites. As a representative work, Wang et al. [107] fabricated MWCNT/TiO_2_ composite photocatalysts using the sol-gel method and the followed heat treatment. In a general approach, before the sol-gel process, the surface of the pristine MWCNTs should be activated by a surface treatment with concentrated nitric acid, or a mixture of concentrated nitric acid and sulfuric acid to several functional groups can be grafted on the walls of the CNTs so that they can act as reaction sites to facilitate the contact between the semiconductors and the CNTs. By virtue of their good conductivity and large surface area, the introduced MWCNTs played a critical role in enhancing the charge transport and light adsorption capacity. Therefore, the obtained MWCNT/TiO_2_ composite photocatalysts exhibited remarkable enhancement in the visible-light activity compared with pristine TiO_2_. Subsequently, Xu et al. [108] reported a wet impregnation method for the fabrication of MWCNT/TiO_2_ composite photocatalysts, which was even simpler than the above-mentioned sol-gel method. In this study, the acid-activated MWCNTs were uniformly mixed with titanium tetrasiopropoxide in an anhydrous ethanol solution in a certain ratio, and the obtained suspensions were aged for a specific time with continuous stirring. Then, the suspensions were sufficiently dried and calcined in air to obtain the MWCNT/TiO_2_ composite photocatalysts, which was efficient in the photocatalytic degradation of organic compounds in water. Unlike the traditional sol-gel method, this method involved a simple hydration/dehydration process and was therefore slightly more efficient. However, the adapted inorganic precursors may be limited, thus this method may be not suitable for other semiconductors. Consequently, the synthesis method for the CNT-based composite should be flexible and combinable so that a variety of novel semiconductors can be loaded with the CNTs. More recently, Gopannagari et al. [109] successfully synthesized a composite photocatalysts based on platinum (Pt), CdS, and surface-modified CNTs. Before loading the CdS nanorods, the employed MWCNTs were twice under surface modifications to produce various functional groups to capture metal particles, therefore the strength of interfacial interactions could be well ensured. Moreover, the final composite of CdS nanorods and the surface modified CNTs just rely on a simple sonication and stirring process, which was quite easy to operate and energy-saving. After comprehensively evaluating the photocatalytic water splitting performance of corresponding composite photocatalysts, they found that the ternary photocatalysts of Pt, CdS, and ascorbic acid modified CNTs(Pt–Af–CNT/CdS)exhibited superior photocatalytic activity with about 48- and 3.5-fold enhancement compared to that of pristine CdS and Pt/CdS (Figure 5a). They claimed that this significant enhancement was attributed to the improved deposition of Pt nanoparticles on the surface of modified CNTs and the interaction with CdS nanorods, which enhanced the electron–hole pair separation and retard recombination process of the composites (Figure 5b). 

**Figure 6:**
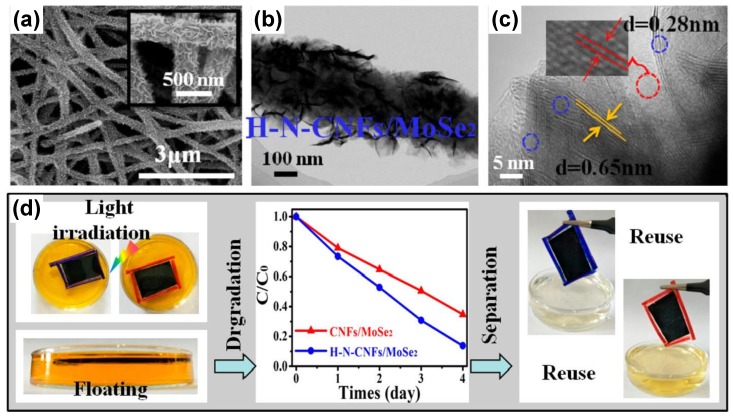
(**a**) SEM images, (**b**,**c**) Transmission electron microscopy (TEM) images of H–N–CNF/MoSe_2_ heterojunctions. (**d**) Digital photographs demonstrating the self-supporting photocatalysis application of H–N–CNF/MoSe_2_ and CNFs/MoSe_2_. Adapted with permission from Reference [124]. Copyright (2018) American Chemical Society.

Apart from CNTs, CNFs are also attractive for the development of next-generation photocatalysts. Similar to CNTs, CNFs also possess the appreciable properties of conductivity, large surface area, and good physicochemical stability. However, the aspect ratio of CNFs could be much larger than that of CNTs, thus endowing CNFs with much better assemblability [110]. In addition, the porous structure and composition of CNFs can be facilely regulated by employing the appropriate synthesis techniques [111]. The most commonly reported fabrication methods for CNFs are the chemical vapor deposition method [112,113,114], the floating catalyst method [114,115,116,117], and the electrospinning method [118,119,120]. Among which, the electrospinning method has become a powerful and highly versatile technique for the fabrication of CNFs because of its easy operation, low cost, and good feasibility for a wide range of carbon precursors [27,30,111,121,122]. Consequently, electrospun CNFs have attracted considerable research attention in the photocatalysis field, and many outstanding studies have been reported. For example, Mu et al. [123] fabricated an In_2_O_3_ nanocube/CNF composite photocatalyst by combining electrospinning with the solvothermal method. In that study, polyacrylonitrile (PAN) was used as the carbon precursor to prepare PAN nanofibers via electrospinning the solutions, and after subsequent carbonization, CNFs with good fibrous structures were obtained. The as-prepared CNFs were used as substrates for the growth of In_2_O_3_ nanocubes on their surface by the solvothermal treatment method. The obtained In_2_O_3_/CNF composite exhibited remarkable improvement in the visible-light photocatalytic degradation performance due to the enhanced charge separation efficiency resulting from the synergistic effect between In_2_O_3_ and the CNFs. Moreover, with a good combination of electrospun CNFs, a variety of novel CNFs supported composite photocatalysts derived from different semiconductors could also be facilely synthesized. Our group fabricated Ag–ZnO-decorated CNFs by combining electrospinning with hydrothermal treatment. Similarly, carbonized PAN electrospun nanofibers were fabricated as the substrate and subsequently, an aqueous suspension containing Ag and ZnO precursors and the CNFs were subjected to hydrothermal treatment [111]. The photocatalytic performance of the obtained Ag–ZnO NPs/CNF composite was considerably improved due to the decreased recombination rate of the photogenerated electrons and holes as well as the increased rate of electron transport, high mobility of the charge carriers, and excellent adsorption capacity of the CNFs [111]. More recently, Yang et al. [124] fabricated nitrogen-doped CNFs with hierarchical structures, namely H–N–CNFs, by combining electrospinning and an in situ polymerization method. Subsequently, a hydrothermal method was employed to construct molybdenum diselenide (MoSe_2_) nanosheets on the surface of the obtained H–N–CNFs to form H–N–CNF/MoSe_2_ heterojunctions (Figure 6a–c). Although several manufacturing processes were involved in this approach, the fibrous structure of the CNFs was well preserved. The H–N–CNF/MoSe_2_ composite exhibited an obvious improvement in the photocatalytic performance upon full-spectrum irradiation. This phenomenon occurred because the hierarchical nanostructures of the H−N−CNF supports allow for more efficient interfacial contact between MoSe_2_ and the CNFs so that fast interfacial charge transfer could be achieved. Consequently, the CNF-based composite photocatalyst showed good recyclability owing to the excellent mechanical properties of the CNFs and the higher aspect ratio compared with that of the CNTs (Figure 6d). 

### 3.4. Graphene 

Graphene is a widely concerned two-dimensional (2D) carbon nanomaterial with great potential for numerous applications. In general, graphene has a unique layered structure comprising sp^2^- bonded carbon atoms in a hexagonal lattice, and the one-atom-thick carbon layers endow it with desirable features such as excellent mechanical properties, intriguing heat and electron conductivity, large surface area, and robust physicochemical stability [125,126]. Moreover, graphene is a semimetal with a small degree of overlap between the VB and CB [127], making it a promising candidate for application in photocatalysis. 

**Figure 7:**
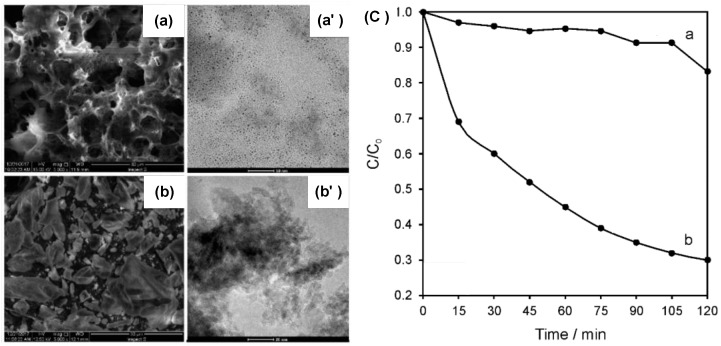
(**a**,**b**) SEM, (**a’**,**b’**) TEM of pristine GQDs and NiAl_2_O_4_/GQDs composite, respectively. (**c**) Photodegradation performances of NiAl_2_O_4_ (line a) and NiAl_2_O_4_/GQDs composite (line b) over the Rhodamine B (RhB). Adapted with permission from Reference [136]. Copyright (2019) MDPI AG.

Combining graphene with certain semiconductors to form a graphene/semiconductor composite photocatalyst system is a facile and commonly used strategy to produce a high-performance photocatalyst [128,129,130,131,132,133]. For example, Zhang et al. [134] once reported a facile approach for the fabrication of a graphene/TiO_2_ composite. Accordingly, graphene oxide (GO) and commercial TiO_2_ powders (P25) were well dispersed in a mixed solution of water and ethanol to prepare a homogeneous suspension, which was hydrothermally treated and dried to obtain the graphene/TiO_2_ composite. As demonstrated in this work, the TiO_2_ nanoparticles were well anchored on the surface of the graphene, and the composite photocatalyst exhibited superior photocatalytic performance when compared with the pure P25 and the CNT/P25 composites, owing to the 2D planar structures of graphene which enhanced the adsorption capacity of the organic compounds and facilitated charge transport [133]. Because that study confirmed the significant contribution of graphene in enhancing the photocatalytic activity of TiO_2_, many graphene-based composite photocatalysts with other semiconductors have been developed. Herein, we discuss some representative studies of newly developed semiconductors. For example, Meng et al. [135] recently reported the fabrication of a novel three-component composite photocatalyst with a designed Z-scheme heterojunction for CO_2_ reduction. In this composite system, a metal−organic framework, UiO–66–NH_2_, and oxygen-defective ZnO (O–ZnO) were used as the photocatalysts, while reduced graphene oxide (rGO) acted as the substrate and electron mediator. A facile solvothermal method was employed to synthesize a composite system of these three components. As expected, the obtained composite photocatalysts exhibited excellent photocatalytic activity because the photogenerated electrons in the CB of O–ZnO could be effectively transferred to rGO before recombination with the holes from the VB of UiO–66–NH_2_, therefore, the charge separation efficiency of the composite system was remarkably enhanced. Recently, the graphene quantum dots (GQDs), which are nanoscale fragments of graphene, have also been widely used to composite with semiconductors to improve the photocatalytic performance of semiconductors with several unique properties of size-dependent luminescence, extended π-electron system, discrete electronic levels, and relative low cost for synthesis. For example, Regulska et al. [136] once prepared a novel NiAl_2_O_4_/GQDs composite via a facile co-precipitation method. As shown in Figure 7, they found that the introduced GQDs could effectively prolong the recombination of electron–hole pairs, enhance the harvest of sunlight, and improve the adsorption of pollutants. Consequently, the photocatalytic activity of the obtained composite under the irradiation of solar light toward various organic pollutants was superior to that of pristine NiAl_2_O_4_. 

**Figure 8:**
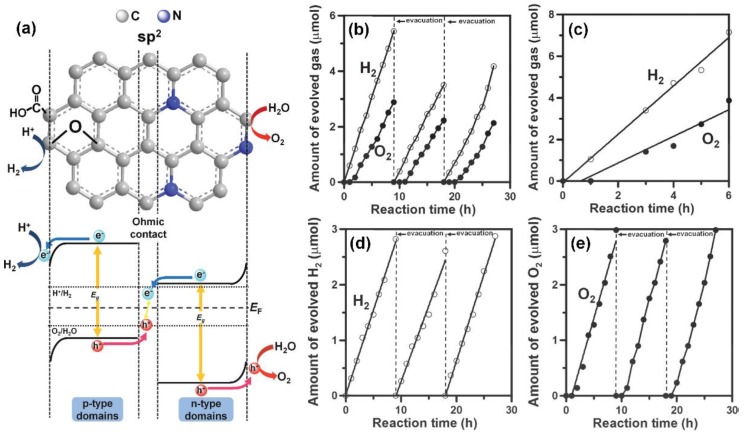
(**a**) Schematic illustration of the configuration and energy diagram for a NGO–QD photochemical diode. Visible light induced photocatalytic water splitting performance over different photocatalysts: (**b**) 1.2 g of NGO–QDs, (**c**) 0.5 g of Rh_2−y_ Cr_y_O_3_/GaN:ZnO, (**d**) 1.2 g of GO–QDs, (**e**) 1.2 g of NH_3_–NGO–QDs. Adapted with permission from Reference [138]. Copyright (2014) Wiley.

Besides, GO could also be utilized for the synthesis of photocatalysts. Our group recently reported the fabrication of a GO/BiOCl/PAN composite by a combination of the solvothermal and electrospinning methods. The composite photocatalyst had a fibrous structure and the presence of GO led to a nearly three-fold enhancement of the photocatalytic degradation activity of the organic pollutants in water, as compared with that of the pristine BiOCl/PAN fibers [137]. Moreover, graphene can be directly used as a photocatalyst. For example, Yeh et al. [138] reported the synthesis of nitrogen-doped graphene oxide quantum dots (NGO–QDs). In their study, GO was pretreated with NH_3_ to incorporate nitrogen elements into the structure of graphene to obtain nitrogen-doped graphene oxide (NGO). Subsequently, NGO was used as a precursor to prepare NGO–QDs via a modified Hummer’s method. Then, the photocatalytic performance of the obtained NGO–QDs under irradiation with visible light was investigated. As shown in Figure 8a, the nitrogen atoms introduced into the graphene frame led to the formation of p–n type photochemical diodes in the NGO–QDs, resulting in n-conductivity. Meanwhile, the presence of oxygen on the graphene surface resulted in p-conductivity. As a result, the NGO–QDs exhibited intriguing visible-light photocatalytic water splitting performance compared with the other photocatalysts (Figure 8b–e). Moreover, the meta-free characteristics of the NGO–QD catalyst make it more sustainable from environmental and economic viewpoints. To summarize, the presence of graphene favored the design and synthesis of novel photocatalysts, which is one of the most promising developmental directions for photocatalysts. 

### 3.5. Fullerene

**Figure 9:**
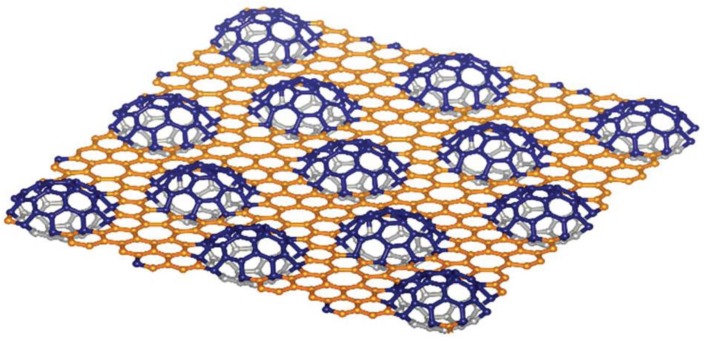
Schematic illustration showing the structures of various bubble-wrap allotropes. Adapted with permission from Reference [141]. Copyright (2018) Royal Society of Chemistry.

Considering that there are many types of C allotropes, some other carbon nanomaterials, such as fullerene (C_60_), could also be utilized as an effective component for the synthesis of novel photocatalysts because of its unique electronic properties [22]. C_60_ is an excellent electron acceptor that can accept six electrons owing to its relatively low-energy LUMO (lowest unoccupied molecular orbital) and high state degeneracy [139]. Therefore, coupling C_60_ with various semiconductors to form C_60_/semiconductor composite photocatalysts has been well studied. For example, Li et al. [39] synthesized a C_60_/Bi_2_TiO_4_F_2_ composite photocatalyst via a facile solvothermal method for the visible light photocatalytic degrading of organic pollutants in water. They demonstrated that C_60_ clusters were well dispersed on the surface of Bi_2_TiO_4_F_2_ to form strong heterojunctions. Therefore, the electron transfer rate and the charge carrier separation efficiency of the photocatalyst were significantly enhanced and C_60_ could further improve the light adsorption capability of the C_60_/Bi_2_TiO_4_F_2_ composite. As a result, the obtained composite photocatalyst exhibited remarkably enhanced activity under visible-light irradiation. On the other hand, the fullerene derivatives could also be utilized to improve the photocatalytic performance of semiconductors. For example, Echegoyen et al. [140] recently explored the application of porphyrin/phthalocyanine fullerene complexes in a photocatalytic system with TiO_2_. In their study, the bis(4-pyridyl)pyrrolidinofullerene was used as a dual ligand for a supramolecular approach to attach porphyrins and phthalocyanines on the surface of TiO_2_ to make it sensitized. The synthesis of the composite was based on a simple sol-gel method. As expected, the obtained composite exhibited intriguing photocatalytic activity under the irradiation of solar light, which was attributed to the enhancement of electron transfer to the CB of the corresponding semiconductor. More recently, Liu et al. [141] demonstrated a facile strategy to fabricate a novel 2D carbon material that combined the structures of fullerene and graphene by embedding bubble carbon structures in the graphene lattice (Figure 9). They claimed that by regulating the percentage of sp^3^ carbon and the strains caused by the curvatures of the bubbles, a tunable electronic structure ranging from metallic to semiconducting with sizable gaps can be created in the material. Therefore, this method is a promising tool to create novel carbon-based photocatalysts. 

### 3.6. g-C_3_N_4_

**Figure 10:**
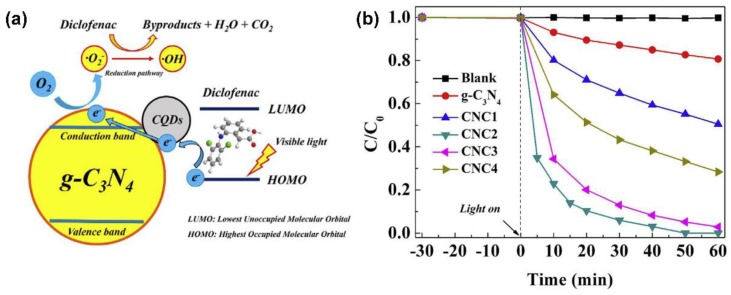
(**a**) Schematic illustration showing the visible light-induced photocatalytic degradation of organic compounds over CQD-modified g-C_3_N_4_. (**b**) Photocatalytic degradation kinetics of diclofenac by different photocatalysts. Adapted with permission from Reference [155]. Copyright (2019) Elsevier.

Recently, the development of metal-free photocatalysts has become a very hot research topic. Unlike traditional metal semiconductors, g-C_3_N_4_ is a polymeric semiconductor that only consists of carbon and nitrogen elements and has a graphite-like layered structure [32]. This unique structure endows g-C_3_N_4_ with intriguing properties such as a small bandgap, good thermal stability, and easy fabrication, all of which make it a promising candidate for the design and fabrication of highly efficient photocatalysts [20,142,143]. Since Wang et al. [144] first employed g-C_3_N_4_ for the visible-light photocatalytic production of H_2_, many g-C_3_N_4-_based photocatalysts have been developed. Additionally, several materials engineering strategies have been invented to further enhance the photocatalytic activity of g-C_3_N_4_. Zhang et al. [145] reported a simple and low-cost method to improve the photocatalytic performance of g-C_3_N_4_. They used sacrificial templates to create pores inside the g-C_3_N_4_ bulk to increase its surface area and porosity so that the mass transfer ability and photocatalytic activity of the tailored g-C_3_N_4_ could be further enhanced. However, this method only focuses on the morphological regulation of pristine g-C_3_N_4_, and the critical problem of fast recombination of charge carriers in g-C_3_N_4_ has not been solved. Therefore, some other approaches focusing on optimization of the bandgap or the charge carrier separation efficiency of g-C_3_N_4_ were proposed, such as heteroatom doping [146,147,148], dye sensitization [149,150,151], and coupling with other semiconductors [152,153,154]. In this review, we will briefly discuss the fabrication and tailoring of the photocatalytic activity of g-C_3_N_4_ based on carbon materials, as carbon is one of the most abundant elements on the earth and is environment-friendly. Recently, Liu et al. [155] reported the fabrication of a new kind of carbon quantum dots (CQD) modified porous g-C_3_N_4_ composite (CNC) photocatalyst via a simple polymerization method. In this method, the as-prepared CQDs were mixed with urea in a designed ratio, followed by polymerization and calcination processes to obtain CNCs. Subsequently, they investigated the photocatalytic activity of the CNCs, and the results showed that the CNCs exhibited almost 15 times higher degradation kinetics toward the diclofenac than that of pure g-C_3_N_4_. This trend was attributed to the improved separation of charge carriers and the tuned band structure, with the CQDs attached on the surface of g-C_3_N_4_ (Figure 10)_._ Moreover, other carbon nanomaterials, such as CNTs [156,157,158,159], CNFs [160,161,162], and graphene [163,164,165], could also be coupled with g-C_3_N_4_ to further improve its photocatalytic activity.

### 3.7. Carbon Sponge/Aerogel

**Figure 11:**
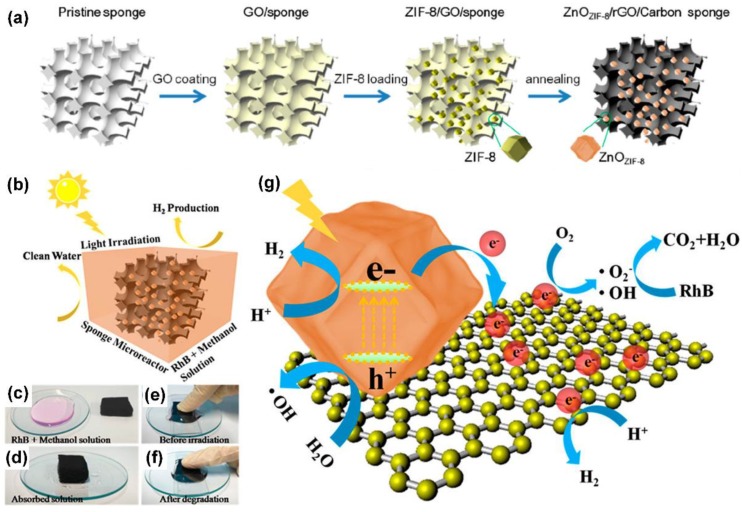
(**a**,**b**) Schematic illustration of the fabrication process and photocatalytic reaction in ZnO nanocages/rGO/carbon sponge (ZRCs)-based microreactor. (**c**–**f**) ZRCs absorption and in-situ photocatalytic degradation of RhB/methanol aqueous solution. (**g**) Schematic illustration demonstrating the mechanism of simultaneous sunlight-driven photocatalytic degradation of RhB and H_2_ production. Adapted with permission from Reference [167]. Copyright (2018) American Chemical Society.

With the rapid development of photocatalysts based on various nanomaterials, some critical issues for practical applications have emerged, including the strong coacervation of nanoscale materials that increases the complexity and cost of the separation of photocatalysts from the reaction system while decreasing the service durability [166]. Recently, the fast development of 3D open-cell materials (e.g., aerogels and nanosponges) provided a new idea for constructing photocatalytic reactors to improve the recyclability and service ability of photocatalysts by anchoring them on the surface of carbonaceous substrates with 3D open-cell structures. The benefits of 3D open-cell substrates for photocatalysis are as follows: (1) open-cell 3D frameworks can provide a more valid surface for the loading of photocatalysts; (2) the good connectivity of pores and the high porosity can ensure the fast transport of reactants; (3) 3D monolithic photocatalysts can be well collected and separated from the reaction system. For example, Su et al. [167] employed a commercial melamine foam (MF) as the support and coated GO on its surface to provide a larger number of binding sites for the functional materials. One kind of metal organic frameworks (MOFs), namely ZIF-8 was chosen as the template for the synthesis of a ZnO nanocage. Then, by using a simple dipping-pyrolysis method, a 3D photocatalytic micro-reactor based on the ZnO nanocages/rGO/carbon sponge (ZRCs) was fabricated (Figure 11a). The obtained 3D photocatalytic micro-reactor exhibited excellent adsorption and intriguing photocatalytic degradation and H_2_ production performance as well as a facile recyclability (Figure 11b–f). The rGO and carbonized MF frameworks not only acted as a conducting medium to facilitate the separation of photogenerated electron−hole pairs but also effectively improved the solar-light absorption capacity of the composite, resulting in a promising enhancement in the photocatalytic activity (Figure 11g). Besides sponges, aerogels based on graphene are also considered highly promising candidates for the synthesis of 3D monolithic photocatalysts. Recently, Hu et al. [168] reported a facile method for the fabrication of a functional aerogel based on perylene imide (PI)-modified g-C_3_N_4_ and GO. Accordingly, PI-modified g-C_3_N_4_ and GO were sufficiently mixed and subjected to hydrothermal treatment, followed by freeze-drying to obtain the composite aerogel. The aerogel showed excellent photocatalytic activity for the decomposition of NO gas under visible-light irradiation, which was attributed to the strong visible-light absorption capacity, good charge transport properties, and large specific surface area of the as-synthesized aerogel.

## 4. Summary and Perspectives

To fulfill the requirements of various photocatalytic processes, several novel and highly efficient photocatalysts have been invented with the development of synthesis technologies. This review provided a summary on the design and synthesis of carbonaceous photocatalysts for various applications of environmental remediation and energy conversion. The highlighted carbonaceous photocatalysts in this review mainly derive from activated carbon, carbon dots, carbon nanotubes/nanofibers, graphene, fullerene, g-C_3_N_4_, and carbon sponges/aerogels. After a comprehensive evaluation of the corresponding synthesis methods and the photocatalytic performances of the above-mentioned carbonaceous photocatalysts, it could be concluded that carbonaceous materials, especially the nanocarbon materials, exhibit intriguing ability in enhancing the photocatalytic performance of various photocatalysts. Although there are some differences in the morphologies and instincts of the employed carbonaceous materials, the mechanism for the enhancement of photocatalytic activity could be proposed as the following aspects: 1) with the introduction of a carbon component, a great number of heterojunctions can be generated in the composite of carbon/semiconductors, which effectively trap the generated electrons to suppress the recombination of electron–hole pairs [29,48,169,170]. 2) Carbonaceous materials are capable of enhancing the visible light absorption capacity and the electron transportation and separation efficiency of the composite could be significantly improved due to the good conductivity of carbon [171,172,173,174]. 3) Owing to the high surface area of carbon phase, many more reaction sites are presented and the adsorption capacity of reactants are significantly enhanced, therefore the availability of the photocatalysts could be improved. 4) The excellent chemical/physical properties of carbon could ensure a good utilization stability of the carbonaceous photocatalysts. Consequently, the carbonaceous photocatalysts have become a promising candidate for the practical photocatalytic applications and even more new carbonaceous photocatalysts will be invented in the near future. 

Although several great advancements have been made in the development of carbonaceous photocatalysts, there are still many critical limitations which need to be overcome to promote their practical applications. Herein, we provide a brief summary of the existing challenges of various carbonaceous materials and some plausible perspectives on the development of carbonaceous photocatalysts, which can be summarized by the following points: 1) in most case, semiconductors are loaded on the surface of a carbon matrix, the interface contact of carbon phase and semiconductors are not so intimate, resulting in a limited bonding strength and electron transport ability. Therefore, the basic properties of the carbonaceous photocatalysts, including the bandgap energy, charge separation efficiency, and light absorption capacity of the photocatalysts, could be further improved by optimizing the compatibility of carbon phase and the semiconductors. More attention could be paid to the modification of carbon matrix via heteroatom doping (e.g., O, N, P) to improve the surface activity of carbon and providing more active sites for the chemical anchor of semiconductors. 2) The nanostructured carbons, such as the carbon dots, CNT, and graphene, are efficient in enhancing the photocatalytic activity of the composite photocatalysts, however the powdered morphology in the macroscale make them quite difficult to recycle after the reaction, and a secondary pollution may be generated if the powdered photocatalysts are leaked by accident. Therefore, the 3D monolithic photocatalysts are preferred and a few 3D carbonaceous photocatalysts have been developed. However, the mechanical strength of 3D monolithic substrates and the bonding strength of the semiconductors with those carbonaceous substrates need to be further enhanced to ensure good stability and more attention could be paid to the direct assembly of the carbon/semiconductor composite via the 3D casting techniques. 3) Carbon is an earth-abundant element, however, the carbonaceous materials just act as substrates and co-catalysts in the composite system due to their poor photocatalytic activity, while most of the “leading role” semiconductors are limited. Therefore, it is very important to reveal the mechanism of carbon for enhancing the photocatalytic performance, which is not so clear now. By understanding the enhancing mechanism, the utilization of the semiconductors could be more efficient, thus the cost of composite photocatalysts could be reduced, which is crucial for the practical applications. For the study of photocatalytic mechanisms of various carbonaceous photocatalysts, more attention could be paid to establishing a comparable testing standard at first, and the existing fundamentals, such as p–n junctions, heterojunctions, Z-scheme systems, Schottky junctions, and phase and facet junctions, should be comprehensively considered. 4) The design of carbonaceous photocatalytic materials should sufficiently consider their applications and commercial benefits, and explore environmentally friendly and energy-saving synthesis strategies. 

In summary, carbonaceous materials have been proven to be of significant importance for the design and synthesis of advanced photocatalysts and a great deal of carbonaceous photocatalysts have been developed. The impressive advancements of the carbonaceous photocatalysts open an avenue for exploring the application of carbon in the area of photocatalysis. Although there are still several challenges, the great merits of various carbon materials and their tunable properties offer numerous opportunities for the development of novel carbonaceous photocatalysts with intriguing specific functions, and good practical performance both in scientific and industrial communities. Finally, we anticipate this review could provide some guidance for the design and synthesis of the next generation of carbonaceous photocatalysts for different applications. 

## Figures and Tables

**Table 1:** Summary of representative carbonaceous photocatalysts synthesis approaches, and their applications.

Photocatalyst	Synthesis Method	Main Precursor	Morphology	Applications	Irradiation Light	Ref.
TiO_2_/AC	Ultrasonic-assisted sol-gel	Titanium(IV) n-butoxide, commercial AC	Powder	Reduction of Cr(VI)	PLS-SXE 300 Xe lamp (UV, λ > 400, λ > 420 nm)	[63]
BiOI/AC	Hydrothermal	Bismuth nitrate, potassium iodide, commercial AC	Powder	Degradation of RhB aqueous solution	500 W Xe lamp	[67]
ACF/Bismuth oxyhalides (BiOX, X = Cl, Br)	Solvothermal	Bi(NO_3_)_3_·5H_2_O, 1-hexadecyl-3-methylimidazoli-um bromide, 1-hexadecyl-3-methylimidazoli-um chloride	Fiber	Degradation of RhB and 2,4-Dichlorophenol aqueous solution	300 W Xe lamp	[58]
TiO_2_–MnTiO_3_/HACF	Biotemplated method	Kapok, bis (ammonium-lactato) titanium-dihydroxide, Mn(NO_3_)_2_	Fiber	Degradation of methylene blue (MB) aqueous solution	500 W Xe lamp	[35]
BiVO_4_/ACF	Hydrothermal immobilization	Bi(NO_3_)_3_·5H_2_O, NH_4_VO_3_	Fiber	Degradation of reactive Black KN–B aqueous solution	1000 W Xe lamp	[79]
CdS/CDs	Solvothermal, hydrothermal	Sulfur powder, CdCl_2_·2.5H_2_O	Powder	Water splitting	300 W Xe lamp	[85]
Carboxyl-functionalized CDs	Microwave irradiation	Dextrose, urea	Powder	Oxidation of alkyl benzenes	60 W white LED lamp	[86]
MWCNT/TiO_2_	Impregnation	Titanium tetrasiopropoxide, MWCNTs	Powder	Degradation of benzene in the gas phase	Four 4 W UV lamps	[108]
Pt-ascorbic acid modified CNT/CdS	Acid refluxing, adsorption, chemical reduction	L-ascorbic acid, Chloroplatinic acid solution, cadmium acetate, MWCNTs	Powder	Water splitting	150 W Xe lamp	[109]
In_2_O_3_/CNFs	Electrospinning, solvothermal	PAN, In(NO_3_)_3_·5H_2_O, CO(NH_2_)_2_	Fiber	Degradation of RB, methyl orange (MO) aqueous solution	150 W Xe lamp with a UV cut-off filter (λ > 420 nm)	[123]
MoSe_2_/HN–CNFs	Electropsinning, in situ polymerization, hydrothermal	PAN, Na_2_MoO_4_·2H_2_O, Se	Fiber	Degradation of RhB, MO, and tetracycline hydrochloride	300 W Xe lamp	[124]
O–ZnO/UiO–66–NH_2_/rGO	Modified Hummers’, solvothermal	Natural graphite powder, Zn(CH_3_COO)_2_·2H_2_O	Powder	Reduction of CO_2_	300 W Xe lamp with a UV cut-off filter (λ > 420 nm)	[135]
NiAl_2_O_4_/GQDs	Pyrolyzing, coprecipitation	Citric acid, ammonium oxalate, Al(NO_3_)_3_·9H_2_O, Ni(NO_3_)_3_·6H_2_O	Powder	Degradation of RhB, MB, quinoline yellow, eriochrome black, phenol, thiram aqueous solution	SUNTEST CPS+ solar simulator	[136]
BiOCl/GO	Solvothermal, electrospinning	PAN, Bi(NO_3_)_3_·5H_2_O, GO	Fiber	Degradation of RhB	Solar simulator (Sun 2000, ABET) with a UV cut-off filter (λ > 440 nm)	[137]
NGO–QDs	Heat treatment in NH_3_, modified Hummers’ method	NH_3_, GO	Powder	Water splitting	300 W Xenon lamp with a UV cut-off filter (420 < λ < 800 nm)	[138]
Bi_2_TiO_4_F_2_/C60	Solvothermal	Bi(NO_3_)_3_·5H_2_O, TiF_4_, C60	Powder	Degradation of RhB	300 W Xe lamp with a cutoff filter (λ > 420 nm)	[39]
Bis(4-pyridyl)pyrrolidinofullerene/TiO_2_	Sol-gel	4-(aminomethyl)pyridine, 4-piridinecarboxaldehyde, buckminsterfullerene, Titanium isopropoxide, porphyrin/phthalocyanine	Powder	Degradation of MB and phenol	A SUNTEST CPS+ solar simulator	[140]
g-C_3_N_4_	Direct pyrolysis	Dicyandiamide, urea	Powder	Degradation of MB	500 W Xe with a cutoff filter (λ > 420 nm)	[145]
g-C_3_N_4_/CQDs	Hydrothermal	Citric acid, urea	Powder	Degradation of diclofenac	300 W Xe lamp with a cutoff filter (λ > 400 nm)	[155]
ZnO/ZIF–8/rGO/Carbon-sponge	Dipping-pyrolysis	Melamine foam, Zn(NO_3_)_2_, dimethylimidazole	3D monolithic	Degradation of RhB aqueous solution	300 W Xe lamp	[167]
g-C_3_N_4_/GO aerogels	Modified hummer’s method, hydrothermal, freeze drying	Dicyandiamide, graphite powder	3D monolithic	Decomposition of NO gas	300 W Xe lamp	[168]
